# Supplementary material for: Bridging the gap: Enhancing HIV care pathways for young key populations in Chad
Source: PLOS Glob Public Health. 2025 Apr 8;5(4):e0003790. doi: 10.1371/journal.pgph.0003790 (PMC11978077; doi:10.1371/journal.pgph.0003790)
Supplement: S1 Fig — (DOCX) [file pgph.0003790.s004.docx]

**S1 Figure. HIV care continuum analysis matrix for Young Key Populations.**


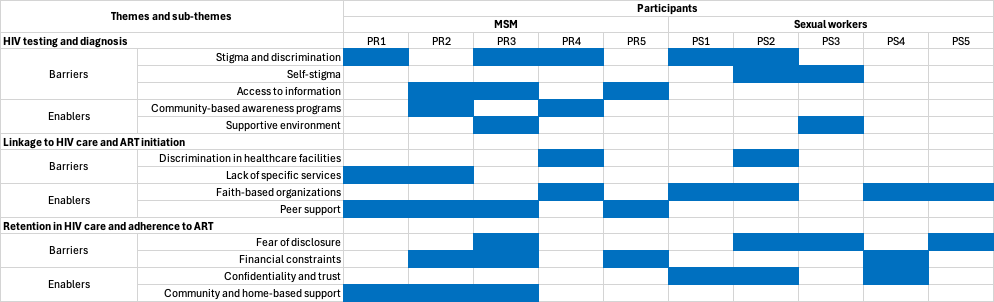


**Notes:** This figure presents a table categorizing barriers and enablers related to HIV across the different themes and sub-themes, as identified by participants. The themes are organized across the HIV care continuum: HIV testing and diagnosis, linkage to HIV care and ART initiation, retention in HIV care and adherence to ART. Participants are grouped into two groups: MSM (PR1, PR2, PR3, PR4, PR5) and sexual workers (PS1, PS2, PS3, PS4, PS5). The colored cells in the table represent the specific themes and sub-themes identified by each participant.
