# Supplementary material for: Bridging the gap: Enhancing HIV care pathways for young key populations in Chad
Source: PLOS Glob Public Health. 2025 Apr 8;5(4):e0003790. doi: 10.1371/journal.pgph.0003790 (PMC11978077; doi:10.1371/journal.pgph.0003790)
Supplement: S1 Table — (DOCX) [file pgph.0003790.s005.docx]

**S1 Table. HIV Statistics for Key Populations.**

|  | **Sex workers** | **MSM** | **PWID** | **Prisoners** |
| --- | --- | --- | --- | --- |
| **Population size estimates (#)** | 33 800 | 8 200 | 710 | 5 700 |
| **HIV prevalence (%)** | 13.8 | 3.9 | N/A | 5.2 |
| **ART coverage (%)** | 83.1 | 33.3 | N/A | 91.7 |
| **Condom use (%)** | 84.0 | 56.4 | N/A | N/A |

**Notes:** MSM – men who have sex with men; PWID – people who inject drugs.
