# Supplementary material for: Bridging the gap: Enhancing HIV care pathways for young key populations in Chad
Source: PLOS Glob Public Health. 2025 Apr 8;5(4):e0003790. doi: 10.1371/journal.pgph.0003790 (PMC11978077; doi:10.1371/journal.pgph.0003790)
Supplement: S2 Table — (DOCX) [file pgph.0003790.s006.docx]

**S2 Table. HIV care continuum sample coding for Young Key Populations.**

| **Transcript excerpt** | **Code** | **Subtheme** | **Theme** |
| --- | --- | --- | --- |
| “The issues that lead people like us not to seek HIV care are stigmatization of MSM by society” (PR1, 16 years old) | Stigma and discrimination | Barriers | HIV testing and diagnosis |
| “The service is easy for me to access. The amenities are not the same because private and religious institutions are better organized than the public ones” (PR3, 23 years old) | Supportive environment | Facilitators |  |
| “Specialized HIV care services need to make an effort to support our community and train their health workers to better serve our community” (PR2, 15 years old) | Lack of specific services | Barriers | Linkage to care and ART initiation |
| “I trust the care at the faith-based institution because they treat me with dignity, and that helps me stay committed to my ART medications” (PS5, 20 years old) | Faith-based organizations | Facilitators |  |
| “Sex workers are afraid to continue to seek healthcare services because there is stigma in health services and confidentiality is not guaranteed” (PS3, 22 years old) | Fear of disclosure | Barriers | Retention in care and adherence to ART |
| “When I trust the healthcare worker, I’m more likely to follow their advice and come back for my check-ups” (PS4, 24 years old) | Confidentiality and trust | Facilitators |  |
