# Supplementary material for: Bridging the gap: Enhancing HIV care pathways for young key populations in Chad
Source: PLOS Glob Public Health. 2025 Apr 8;5(4):e0003790. doi: 10.1371/journal.pgph.0003790 (PMC11978077; doi:10.1371/journal.pgph.0003790)
Supplement: Attachment — Submitted filename: Comments for manuscript.docx [file pgph.0003790.s007.docx]

**REVIEWER’S COMMENTS**

I would like to commend the authors for their valuable contribution to this important topic. I hope the following feedback will support the authors in further strengthening the manuscript.

**Abstract**

The abstract summarizes the study well, providing an overview of the research aims, methods, results, and conclusions. However, the abstract could benefit from quantifying certain aspects, such as the number of participants or specific outcomes. Also, the conclusion in the abstract lack specificity in terms of actionable recommendations.

**Introduction**

1. The introduction could benefit from clearer articulation of the research gap. While the general challenges faced by key populations are well-explained, there is less emphasis on what specifically is unknown about young key populations in Chad.
2. More information about the national and regional policies that shape HIV care in Chad, such as legal frameworks, could enhance the contextual background.
3. The literature review could be more comprehensive, especially concerning other countries with similar epidemics to highlight why Chad is a unique case.

**Methods**

The snowball sampling technique is justifiable given the marginalized nature of the populations being studied. I suggest the following comments for improvements:

1. Please provide more information about the selection and training of interviewers. Were they trained specifically to handle the sensitivities related to the participants’ HIV status and sexual identity?
2. The sample size (10 participants) is small, and while qualitative studies do not require large samples, the limited scope (restricted to N'Djamena) may hinder the generalizability of the findings.
3. While snowball sampling is appropriate for hard-to-reach populations, this method could introduce bias by limiting participant diversity. Kindly discuss how this may have impacted their results.
4. The analysis section lacks sufficient detail. While framework analysis is mentioned, it would be beneficial to elaborate on how themes were coded and interpreted. How was intercoder reliability ensured?
5. No mention of whether data saturation was reached. This is critical in qualitative studies to ensure the depth and comprehensiveness of the findings.

**Results**

1. The results focus heavily on barriers, with comparatively less emphasis on facilitators. While the barriers are important, more detailed exploration of successful interventions (e.g., community-based programs) would offer a more balanced view.
2. There is a lack of differentiation in experiences between sex workers and MSM. More attention to how these two groups differs in their experiences with HIV care could enhance the specificity of the findings.
3. The results are geographically limited to N’Djamena. A brief explanation of whether the findings could be generalized to other parts of Chad (e.g., rural areas) would be helpful.

**Discussion**

1. The discussion lacks depth in proposing specific policy recommendations. While reducing stigma is a key finding, how can healthcare workers in Chad be trained to reduce discrimination? What are some practical steps for implementation?
2. Please expand on the role of legal reforms. The discussion briefly mentions laws protecting people living with HIV, but it would benefit from more in-depth analysis of how these laws are or are not being enforced, and what role legal advocacy might play.
3. There is little critical reflection on the study’s limitations, which weakens the discussion. The small sample size and urban-centric focus should be acknowledged more transparently.

**Conclusion**

1. The conclusion could be more specific in its recommendations. How can interventions be tailored to Chad’s unique cultural and socio-economic environment? Providing a roadmap for future research or pilot programs would strengthen the manuscript’s impact.
2. The conclusion could tie back to broader global HIV efforts, such as UNAIDS targets, to underscore the relevance of this study within global HIV care strategies.

**References**

1. The reference list could benefit from more recent studies focusing on Chad specifically. While global and sub-Saharan African data are included, more country-specific references would strengthen the contextual relevance.
2. Some key recent studies on HIV-related interventions in other African countries could be included to provide a more comparative perspective.

**General Comments**

This manuscript makes a valuable contribution to understanding the challenges faced by young key populations in accessing HIV care in Chad. The manuscript could make an even stronger contribution to the field if the following comments are addressed as well:

1. The study only includes MSM and sex workers, leaving out other key populations, such as people who inject drugs (PWID) or transgender individuals, who may face unique challenges in accessing HIV care. Including these groups in future studies would provide a more comprehensive picture of the HIV epidemic among key populations in Chad.
2. While the manuscript provides general recommendations for addressing barriers (e.g., reducing stigma in healthcare settings), these recommendations could be made more specific. For example, outlining precise interventions (e.g., training programs for healthcare providers, targeted financial aid mechanisms) would give practitioners more actionable steps to take.
3. The data availability statement mentions that the data cannot be shared due to ethical restrictions. While this is understandable, it would be beneficial if the authors could share non-identifiable data, such as anonymized transcripts or coding frameworks, to support transparency and reproducibility.
