# Supplementary material for: Bridging the gap: Enhancing HIV care pathways for young key populations in Chad
Source: PLOS Glob Public Health. 2025 Apr 8;5(4):e0003790. doi: 10.1371/journal.pgph.0003790 (PMC11978077; doi:10.1371/journal.pgph.0003790)
Supplement: Attachment — Submitted filename: Response to Reviewers.docx [file pgph.0003790.s008.docx]

*Drs. Catherine Kyobutungi and Madhukar Pai*

Editors-in-Chief

*PLOS Global Public Health*

February 3^rd^, 2025

Dear Editors-in-Chief,

Thank you for your insightful and constructive comments regarding our manuscript. We have taken a great consideration for each of the comments provided. We provide a detailed response to each of the points raised:

1. Abstract
   a. Added the total number of participants and specific outcomes, such as the themes identified.
   b. Revised the conclusion to include specific suggestions, such as scaling up community-based interventions and integrating stigma-reduction training into healthcare systems.
   c. Added author summary immediately after the abstract.
2. Introduction
   a. Streamlined repetitive points to maintain conciseness.
   b. Clarified global vs. regional statistics for HIV burden among key populations.
   c. Condensed discussion of legal barriers with precise references to Chad’s laws.
   d. Emphasized young key populations rather than general youth.
   e. Removed unnecessary examples while preserving key insights.
   f. Added discussion on current pathways to HIV care for the general population vs. young key populations.
   g. Strengthened rationale by explicitly stating the research gap and study objectives.
3. Methods
   a. Justified the sample size and explained data saturation.
      i. Given the exploratory nature of this study, the qualitative approach prioritizes depth over breadth. While quantitative studies require larger sample sizes for generalizability, qualitative research aims to reach thematic saturation, meaning no new themes emerged during analysis. Our sample size of 10 participants was sufficient to achieve this, as additional interviews with MSM and sex workers were unlikely to yield significantly different insights. However, we acknowledge that future research should expand the sample size and include other key populations to capture a broader range of experiences and enhance representativeness.
   b. Added details on interviewer selection, training, and gender-matching process.
   c. Expanded on snowballing sampling limitations and potential bias mitigation strategies.
   d. Clarified the original languages and how translations were handled.
      i. We explicitly stated that interviews were conducted in French and Arabic, and the analysis was performed in the original languages before translation into English. This ensured that linguistic and cultural nuances were preserved, reducing potential loss of meaning in translation. Transcripts were cross-checked by bilingual researchers to verify accuracy and contextual fidelity before integrating findings into the final analysis.
   e. Specified the software used for data analysis.
   f. Elaborated on triangulation and how themes were validated.
   g. Clarified assent and guardian consent for participants under 18.
4. Results
   a. Clarified sample size justification and demographic details.
   b. Streamlined barrier and enabler discussions for conciseness.
   c. Strengthened the description of stigma and discrimination as a recurring barrier.
      i. Emphasized how both healthcare-related stigma and self-stigma create barriers at multiple stages of the HIV care continuum, reinforcing the urgent need for targeted interventions.
   d. Enhanced clarity on the imbalance between barriers and facilitators.
      i. Highlighted that barriers were mentioned more frequently than facilitators, indicating that participants were more likely to discuss challenges rather than enablers.
   e. Ensured better flow and readability.
   f. Revised table 1 to incorporate reviewer’s feedback by making sub-themes self-explanatory, separating stigma from discrimination, and improving clarity.
5. Discussion
   a. Enhanced policy recommendations by incorporating case studies and best practices from other countries.
   b. Integrated long-term, sustainable solutions, such as vocational training, microfinance, and social protection programs, to improve HIV care retention and ART adherence among young key populations.
   c. Linked findings to global HIV targets and strategies, ensuring that Chad’s response is framed within global best practices.
6. Conclusion
   a. Revised the conclusion to include clear, actionable steps tailored to Chad’s socio-economic and legal environment.
   b. Streamlined to reduce verbosity while maintaining clarity, ensuring that recommendations are direct and actionable.
   c. Emphasized how these interventions align with UNAIDS’ 95-95-95 framework, positioning Chad’s response within global HIV prevention and treatment strategies.

We are confident that these revisions have strengthened our manuscript and addressed the concerns raised. We hope that the revised manuscript will now be considered favorably for publication at PLOS Global Public Health.

Thank you once again for your constructive feedback. We are available to address any queries or provide additional information as needed. Please address all correspondence to: [esias_bedingar@g.harvard.edu](mailto:esias_bedingar@g.harvard.edu) or [esias.bedingar@gmail.com](mailto:esias.bedingar@gmail.com).

Sincerely,

Esias Bedingar
